# The agreement of the OMERACT grey-scale ultrasound scoring system for salivary glands and minor salivary gland biopsy results in a single-center cohort of patients with suspected Sjögren’s disease

**DOI:** 10.1007/s00256-025-04885-7

**Published:** 2025-02-10

**Authors:** Nanna Surlemont Schmidt, Viktoria Fana, Stylianos Georgiadis, Uffe Møller Døhn, Lene Terslev

**Affiliations:** 1 Center for Rheumatology and Spine Diseases, Copenhagen Center for Arthritis Research (COPECARE), Rigshospitalet, Copenhagen, Denmark; 2 Department of Clinical Medicine, University of Copenhagen, Copenhagen, Denmark

**Keywords:** Sjögren’s syndrome, Ultrasonography, Biopsy, Salivary glands, Diagnostic imaging

## Abstract

**Objective:**

Salivary gland ultrasound (SGUS) is a non-invasive tool for the evaluation of parenchymal changes related to primary Sjögrens disease (pSjD) with the potential to reduce the need for minor salivary gland biopsies when diagnosing patients with pSjD. The aim was to assess the agreement between SGUS findings and minor salivary gland biopsy results in patients suspected of pSjD.

**Methods:**

All patients referred with a suspicion of pSjD and scheduled for a diagnostic minor salivary gland biopsy were included in the period 2017–2021. All underwent SGUS of the parotid and submandibular glands bilaterally, Schirmer’s test, unstimulated salivary flow, and blood samples including autoantibody analysis. Changes in the four glands were scored 0–3 using a previously developed ultrasound atlas based on the OMERACT SGUS scoring system for pSjD. All biopsies were scored at the same pathology department and a focus score > 1 was indicative of pSjD.

**Results:**

Of 103 patients included, 43 (42%) were diagnosed with pSjD and 40 (39%) fulfilled the 2016 ACR/EULAR classification criteria. Thirty-two (31%) had a positive minor salivary gland biopsy. The sensitivity of SGUS score ≥ 2 in at least 1 gland was 0.59 and the specificity 0.75. The positive predictive and negative predictive values were 0.51 and 0.80, respectively. The agreement slightly improved when combined with an abnormal salivary flow rate or abnormal Schirmer’s test.

**Conclusion:**

In patients with suspected pSjD, we found good agreement between the OMERACT SGUS scoring system and minor salivary gland biopsy; however, SGUS cannot yet fully replace biopsy in the diagnostic setup of SjD.

## Introduction

Sjogren’s disease (SjD) is a chronic, systemic, autoimmune disease characterized by immune-mediated destruction of the exocrine glands, systemic B-cell hyperactivation, and activation of inflammatory cells, particularly affecting salivary and lacrimal glands. Despite primary SjD (pSjD) having a high prevalence, diagnosing remains challenging due to the complexity of symptoms and the lack of specific diagnostic criteria. Diagnosis relies on subjective symptoms, such as dry mouth and dry eyes, along with clinical, serological, histological, and functional parameters that reflect the disease involvement of the salivary and lacrimal glands, and potentially other organs.

The strongest indicators of pSjD are lymphocytic infiltration on minor salivary gland biopsy and the presence of autoantibodies (anti-SSA/Ro). These two features play a central role in the current classification criteria established by the American College of Rheumatology (ACR) and European League Against Rheumatism (EULAR) [1], where a total score of 4 points is required to fulfill the criteria. Both minor salivary gland biopsy positivity and SSA positivity are designated as major criteria, each carrying a weight of 3. The minor criteria consist of functional tests for dryness including ocular staining score, Schirmer’s test (for ocular dryness and tear production), and unstimulated whole saliva flow rate (for oral dryness), each carrying a weight of 1. However, these tests show relatively low sensitivity [2]. While minor salivary gland biopsy has a high specificity and positive predictive value for SjD, the sensitivity may be variable. The biopsy findings can sometimes be inconclusive or even negative, with significant discrepancies in the histological evaluation [3, 4]. Moreover, the biopsy procedure itself carries potential side effects, such as tissue necrosis and pain due to damage of small sensory nerve branches [5].

Ultrasound of the salivary glands (SGUS) has emerged as a non-invasive and reliable method that can assist in the diagnosis of SjD [6]. A systematic review and meta-analysis on the diagnostic accuracy of SGUS in relation to minor salivary gland biopsy found a good sensitivity and high specificity of SGUS for the diagnosis and a meta-analysis for the predictive value of SGUS for positive biopsy result showed a sensitivity of 0.86 and specificity of 0.87 [7]. It has therefore recently been proposed as a minor criterium in the classification criteria, with a weight of 1 [1, 8–10].

Greyscale (GS) ultrasound of the salivary glands allows for rapid assessment of the parenchymal changes and is widely available in routine rheumatological care. Numerous scoring systems have been proposed over the years to assess the degree of morphological changes affecting the generalizability of research findings and hindering the use of SGUS as a research tool in patients with primary SjD. To address this issue, the Outcome Measures in Rheumatology (OMERACT) ultrasound working group developed and validated a GS semi-quantitative consensus-based scoring system for the major salivary glands to ensure consistency among future studies [11]. The scoring system has further demonstrated good sensitivity and excellent specificity for meeting the SjD classification criteria, when a GS score > 2 in at least 1 gland was considered indicative of primary SjD [10, 12, 13]. Furthermore, a recent study suggested that incorporating SGUS into the classification criteria could potentially eliminate the need for biopsies in more than 25% of the patients suspected of having primary SjD [10].

Consequently, there is considerable interest in assessing the concordance between the scoring system and the minor salivary gland biopsy, even though the latter serves as a proxy measure for changes in the major salivary glands. The aim of the current study was (i) to assess the agreement between minor salivary gland biopsy findings and parenchymal changes identified by SGUS in the major salivary glands of patients suspected of primary SjD (construct validity) and (ii) to assess the optimal number of involved glands on SGUS for an agreement with minor salivary gland biopsy results.

## Patients and methods

In this cross-sectional, retrospective study, we included patients referred to the center for Rheumatology and Spine Diseases at Rigshospitalet, Glostrup, in the period 2017 to 2021, suspected of primary SjD and scheduled for a minor salivary gland biopsy. All patients were > 18 years of age. Patients suspected of having primary SjD who did not have a biopsy were excluded from the study.

All underwent SGUS of the parotid and submandibular glands bilaterally, clinical examination, Schirmer’s test, and unstimulated salivary flow rate and had blood samples taken for assessing autoantibody analysis. Schirmer’s test measures tear production by placing a small strip of filter paper under the lower eyelid to evaluate lacrimal gland function. The biopsy was carried out as the last test.

### Ultrasound assessment

The ultrasound examinations were performed by three rheumatologists with > 10 years of experience in musculoskeletal ultrasound and > 8 years of experience in SGUS. A GE Logiq E9 R6 and E10 R3 (Milwaukee, WI, USA) ultrasound machine with an ML6–15 linear array transducer was used for all examinations. The ultrasound performing rheumatologist was blinded to clinical and serological data. The ultrasound examination was performed with the patient in the supine position and included longitudinal and transverse scans of the parotid glands and longitudinal scans of the submandibular glands as previously described [14]. GS images of each gland illustrating the maximal grade of pathology found in the glands were stored in all patients. A score > 2 was defined as pathological for the individual gland [10]. The ultrasound examination took less than 10 min.

Subsequently, the same three rheumatologists scored each gland 0–3 according to the OMERACT semi-quantitative ultrasound scoring system for SjD [11] using a previously developed ultrasound atlas for the OMERACT SGUS scoring system as reference [10]. A consensus score for each gland was obtained.

Two different ultrasound scores for SjD were tested: US score > 2 in at least one gland and US score > 2 in at least two glands.

### Minor salivary gland biopsies

All minor salivary gland biopsies were performed by the Department of Oral Surgery under local anesthesia with lidocaine. Salivary gland samples were evaluated by the Department of Pathology at Rigshospitalet, Copenhagen, using the focus score method and blinded to clinical and serological data. A focus score ≥ 1 was considered indicative of SjD [1].

The study was evaluated by the local ethical committee and did not require ethical approval—assessment number H-20033020.

### Statistics

Descriptive statistics were applied, and data was expressed as mean (standard deviation) or as percentages. Analysis of variance or Kruskal–Wallis tests were used to compare continuous variables between groups. For categorical variables, Chi-squared or Fisher’s exact tests were used to compare proportions between groups. A *p*-value of < 0.05 was considered statistically significant.

For the diagnostic performance of SGUS vs minor salivary gland biopsy discordance, positive predictive value (PPV), negative predictive value (NPV), sensitivity, specificity, accuracy, balanced accuracy, and kappa statistics, kappa values were interpreted according to Landis and Koch as follows: kappa values of 0–0.2 were considered slight agreement, 0.21–0.40 fair agreement, 0.41–0.60 moderate agreement, 0.61–0.80 substantial agreement, and 0.81–1 almost perfect agreement [15].

Receiver operating characteristic (ROC) analysis was performed to evaluate the diagnostic utility of the SGUS scores compared to the gold standard biopsy and optimal cutoff point of the number of involved glands determined by the Youden index which combines the highest sensitivity and specificity. The area under the curve (AUC) and its 95% confidence interval (CI) were also calculated. The AUC was interpreted as follows: no discrimination (0–0.5), poor accuracy (0.5–0.7), fair accuracy (0.7–0.8), good accuracy (0.8–0.9), or excellent accuracy (0.9–1.0) [16].

All statistical analysis was performed in R (version 4.3.0).

## Results

In the 5-year study period, 103 patients referred to the department for suspected SjD were scheduled for salivary gland biopsy as part of the diagnostic setup and were included in the study. Table 1 shows demographic data for the biopsy cohort and between subgroups based on SGUS findings.

**Table 1:** Demographic characteristics of the cohort stratified by ultrasonographic features and minor salivary gland biopsy results

	All	SGUS (≥ 1 gland score ≥ 2)			SGUS (≥ 2 gland score ≥ 2)			MSGB		
		Positive	Negative	*p*-value	Positive	Negative	*p*-value	Positive	Negative	*p*-value
	*n* = 103	*n* = 37	*n* = 66		*n* = 28	*n* = 75		*n* = 32	*n* = 71	
Age (SD)	55.0 (16.1)	53.4 (18.8)	55.9 (14.4)	0.457	54.0 (19.1)	55.4 (15.0)	0.695	57.6 (15.8)	53.8 (16.2)	0.278
Male sex, *n* (%)	13 (13)	2 (5)	11 (17)	0.128	2 (7)	11 (15)	0.506	4 (12)	9 (13)	1
Symptom duration (SD)	1.2 (0.8)	1.2 (0.9)	1.3 (0.8)	0.701	1.1 (0.9)	1.3 (0.8)	0.335	1.3 (0.8)	1.2 (0.8)	0.370

*MSGB* minor salivary gland biopsy, *SD* standard deviation, *SGUS* salivary gland ultrasonography

Patients with a SGUS score > 2 in at least one gland showed significant differences compared to patients with a negative SGUS in the following areas: clinical diagnosis of primary SjD (59% vs 32%, *p* = 0.012), meeting 2016 ACR-EULAR classification criteria (54% vs 30%, *p* = 0.031), positive biopsy (51% vs 20%, *p* = 0.002), and presence of anti-SSB/La antibodies (19% vs 3%, *p* = 0.01). Very similar findings were seen for patients with SGUS score > 2 in at least two glands—see Table 2.

**Table 2:** Clinical and laboratory characteristics of the cohort stratified by ultrasonographic features

	All	SGUS positive (≥ 1 gland score ≥ 2)		SGUS positive (≥ 2 gland score ≥ 2)		MSGB positive	
	*n* = 103	*n* = 37	*p*-value	*n* = 28	*p*-value	*n* = 32	*p*-value
Clinical diagnosis (%)	43 (42)	22 (59)	**0.012**	17 (61)	**0.031**	32 (100)	** < 0.001**
Classification criteria (%)	40 (39)	20 (54)	**0.031**	15 (54)	0.099	31 (97)	** < 0.001**
Anti-SSA/Ro pos (%)	39 (38)	17 (46)	0.292	14 (50)	0.186	16 (50)	0.137
Anti-SSB/La pos (%)	9 (9)	7 (19)	**0.010**	7 (25)	**0.001**	4 (12)	0.454
ANA pos (%)	42 (41)	19 (51)	0.154	16 (57)	0.066	17 (53)	0.135
Abnormal sialometry (%)	66 (64)	24 (65)	1	18 (64)	1	20 (62)	0.998
Abnormal Schirmers’ test (%)	31 (30)	11 (30)	1	10 (36)	0.604	9 (28)	0.951
Biopsy pos (%)	32 (31)	19 (51)	**0.002**	15 (54)	**0.006**	**-**	**-**

*ANA* anti-nuclear antibodies, *MSGB* minor salivary gland biopsy, *SD* standard deviation, *SGUS* salivary gland ultrasonography

For patients with a positive biopsy, there was a statistically significant difference compared to patients with a negative biopsy in obtaining the clinical diagnosis of pSjD (100% vs 15%, *p* < 0.001) and meeting 2016 ACR-EULAR classification criteria (97% vs 13%, *p* < 0.001—see Table 2).

We further found that the optimal cutoff for SGUS gland involvement for the diagnosis of pSjD was a score ≥ 2 in at least one gland—see Table 3.

**Table 3:** Performance of SGUS for positive minor salivary gland biopsy and optimal number of involved glands across subgroups

	*n* = total	AUC (95% CI)	Youden	Sens	Spec	Optimal cut point
All	103	0.70 (0.61–0.78)	0.3402	0.59	0.75	≥ 1
Clinical diagnosis	43	0.68 (0.53–0.81)	0.321	0.59	0.73	≥ 1
Anti-SSA/Ro pos	39	0.66 (0.52–0.80)	0.375	0.38	1.00	4
Anti-SSB/La pos	9	1.00 (1.00–1.00)	1	1.00	1.00	4
ANA pos	42	0.67 (0.54–0.81)	0.3529	0.35	1.00	4
Abnormal sialometry	66	0.72 (0.63–0.83)	0.4109	0.65	0.76	≥ 1
Abnormal Schirmers’ test	31	0.79 (0.62–0.94)	0.5556	0.56	1.00	4

*ANA* anti-nuclear antibodies, *AUC* area under the curve, *SD* standard deviation, *Sens.* sensitivity, *SGUS* salivary gland ultrasonography, *Spec.* specificity

### Agreement between SGUS and minor salivary gland biopsy results

We found the best sensitivity and specificity of SGUS for a positive minor salivary gland biopsy result was achieved with *at least* 1 gland with a score ≥ 2, reaching a sensitivity of 0.59 and specificity of 0.75. The accuracy of SGUS score ≥ 2 in at least one gland to predict a positive minor salivary gland biopsy was fair with an AUC of 0.70 (95% CI 0.61–0.78)—see Table 3.

### Improving the SGUS concordance with minor salivary gland biopsy

We assessed if adding other clinical tests to the SGUS findings could improve the concordance with the minor salivary gland biopsy result, and we found that adding abnormal sialometry to the SGUS findings increased both sensitivity and specificity (0.59 without vs 0.65 with sialometry) and specificity (0.75 without vs 0.76 with sialometry)—see Tables 4 and 5.

**Table 4:** Diagnostic performance metrics for SGUS (≥ 1 gland score ≥ 2) compared with minor salivary gland biopsy across subgroups of the cohort

	*n* = total	SGUS pos *n* (%)	MSGB pos *n* (%)	Disc. *n* (%)	PPV	NPV	Sens	Spec	Acc	Bal. acc	Kappa
All	103	37 (35.9)	32 (31.1)	31 (30.1)	0.51	0.80	0.59	0.75	0.70	0.67	0.33
Clinical diagnosis	43	22 (51.2)	32 (74.4)	16 (37.2)	0.86	0.38	0.59	0.73	0.63	0.66	0.25
Anti-SSA/Ro pos	39	17 (43.6)	16 (41.0)	15 (38.5)	0.53	0.68	0.56	0.65	0.62	0.61	0.21
Anti-SSB/La pos	9	7 (77.8)	4 (44.4)	3 (33.3)	0.57	1.00	1.00	0.40	0.67	0.70	0.37
ANA pos	42	19 (45.2)	17 (40.5)	16 (38.1)	0.53	0.70	0.59	0.64	0.62	0.61	0.22
Abnormal sialometry	66	24 (36.4)	20 (30.3)	18 (27.3)	0.54	0.83	0.65	0.76	0.73	0.71	0.39
Abnormal Schirmers’ test	31	11 (35.5)	9 (29.0)	8 (25.8)	0.55	0.85	0.67	0.77	0.74	0.72	0.41

*Acc.* accuracy, *Bal. acc*. balanced accuracy, *Disc.* disconcordance, *Kappa* Cohen’s kappa value, *MSGB* minor salivary gland biopsy, *NPV* negative predictive value, *PPV* positive predictive value, *Sens.* sensitivity, *SGUS* salivary gland ultrasound, *Spec.* specificity

**Table 5:** Diagnostic performance metrics for SGUS (≥ 2 gland score ≥ 2) compared with minor salivary gland biopsy across subgroups of the cohort

	*n* = total	SGUS pos *n* (%)	MSGB pos *n* (%)	Disc. *n* (%)	PPV	NPV	Sens	Spec	Acc	Bal. acc	Kappa
All	103	28 (27.2)	32 (31.1)	30 (29.1)	0.54	0.77	0.47	0.82	0.71	0.64	0.30
Clinical diagnosis	43	17 (39.5)	32 (74.4)	19 (44.2)	0.88	0.35	0.47	0.82	0.56	0.64	0.20
Anti-SSA/Ro pos	39	14 (35.9)	16 (41.0)	14 (35.9)	0.57	0.68	0.50	0.74	0.64	0.62	0.24
Anti-SSB/La pos	9	7 (77.8)	4 (44.4)	3 (33.3)	0.57	1.00	1.00	0.40	0.67	0.70	0.37
ANA pos	42	16 (38.1)	17 (40.5)	15 (35.7)	0.56	0.69	0.53	0.72	0.64	0.62	0.25
Abnormal sialometry	66	18 (27.3)	20 (30.3)	18 (27.3)	0.56	0.79	0.50	0.83	0.73	0.66	0.34
Abnormal Schirmers’ test	31	10 (32.3)	9 (29.0)	7 (22.6)	0.60	0.86	0.67	0.82	0.77	0.74	0.47

*Acc.* accuracy, *Bal. acc*. balanced accuracy, *Disc.* disconcordance, *Kappa* Cohen’s kappa value, *MSGB* minor salivary gland biopsy, *NPV* negative predictive value, *PPV* positive predictive value, *Sens.* sensitivity, *SGUS* salivary gland ultrasound, *Spec.* specificity

The AUC reached 0.72 (95% CI 0.63–0.83) when adding abnormal sialometry with an optimal cutoff point for SGUS score ≥ 2 in at least 1 gland—see Table 3.

When adding abnormal Schirmer’s test to the SGUS findings, the sensitivity (0.59 without vs 0.67 with abnormal Schirmer’s test) and specificity (0.75 without vs 0.77 with abnormal Schirmer’s test) increased (Tables 4 and 5). Here, the AUC was 0.79 (95% CI 0.63–0.83), and the optimal cutoff point for SGUS score was ≥ 2 of all 4 glands (Table 3).

Adding a positive anti-SSA/Ro to the SGUS findings, neither the sensitivity nor specificity was improved and did not increase the performance of SGUS for a positive minor salivary gland biopsy. A similar trend was observed when adding a positive ANA to the SGUS findings (Tables 4 and 5).

Adding the criteria of positive SSB/La considerably increased the sensitivity, PPV, and NPV but the specificity and accuracy decreased for the performance of SGUS in both one or more and two or more glands. However, the number of patients in this group was markedly low (Tables 4 and 5).

## Discussion

This study evaluates the agreement between SGUS parenchymal changes and minor salivary gland biopsy results in 103 patients suspected of having primary SjD. We found that an SGUS score ≥ 2 in at least one gland had a sensitivity of 0.59 and specificity of 0.75 for a positive biopsy result. The optimal number of involved glands on SGUS to predict a positive minor salivary gland biopsy (and for the diagnosis of pSjD) was found to be at least one gland, in line with previously published data [10] and achieved a fair accuracy (AUC 0.70, 95% CI 0.61–0.78). The sensitivity improved when combining positive SGUS with abnormal sialometry or abnormal Schirmer’s test, but the specificity remained almost unchanged. Our data support the construct validity of the scoring system and reinforce the proposal of adding SGUS as an additional criterion to the 2016 ACR/EULAR classification criteria, rather than replacing the biopsy item, thereby providing clinicians with more tools for patient assessment [9, 10, 14, 17].

Our findings are partly in line with a recent study assessing the diagnostic accuracy of the OMERACT scoring system in a comparable cohort of 132 patients with sicca syndrome. They reported a lower sensitivity of 0.32 and a higher specificity of 0.97 of SGUS for a positive minor salivary gland biopsy, though with a similar AUC of 0.74 [12]. They applied, however, a sum score for all four glands independent of the grade thereby including also normal scores which may have impacted the results. Other studies applying other SGUS scoring systems have reported higher accuracy for predicting a minor salivary gland biopsy [18].

We chose minor salivary gland biopsy for assessing the agreement with SGUS as it is a part of the classification criteria for pSjD [1], but minor salivary gland biopsy is a proxy measure for the changes in the major salivary glands. A study assessing the agreement between SGUS and parotid gland biopsy has found an even better performance of SGUS in predicting a positive parotid gland biopsy result [5]. Parotid gland biopsy can be repeated in the same gland, may cause less long-term morbidity, and may be comparable to minor salivary gland biopsy in diagnostic sensitivity, specificity, and histopathology [19–22].

We investigated if combining SGUS findings with minor items of the classification criteria would improve the accuracy of a positive biopsy result, and we found that adding abnormal sialometry or abnormal Schirmer’s test to the SGUS findings increased sensitivity but with almost identical specificity. This is supported by a recent study using the OMERACT SGUS scoring system where an agreement was found between hyposalivation and SGUS score > 2 [23].

We did not find any improvement in the SGUS performance for a positive minor salivary gland biopsy when adding anti-SSA/Ro to the test. However, the combination of positive SGUS results and positive anti-SSA/Ro antibodies has previously been shown to be highly predictive for primary SjD [5] and the opposite case to rule out a SjD diagnosis [24]. This association should be further explored in larger cohorts as this could potentially reduce the need for minor salivary gland biopsies in selected patients.

In patients with suspected primary SjD, the OMERACT SGUS scoring system demonstrated fair accuracy with minor salivary gland biopsy results with slight improvement when combined with abnormal sialometry or Schirmer’s test. Although SGUS cannot fully replace biopsy in diagnosing pSjD, it has the potential to reduce the need for biopsies and provides valuable diagnostic support, particularly when used alongside other tests, supporting the utility in clinical practice.

## Data Availability

The datasets used and/or analyzed during the current study are available from the corresponding author on reasonable request.
